# Senecavirus A Enhances Its Adaptive Evolution via Synonymous Codon Bias Evolution

**DOI:** 10.3390/v14051055

**Published:** 2022-05-16

**Authors:** Simiao Zhao, Huiqi Cui, Zhenru Hu, Li Du, Xuhua Ran, Xiaobo Wen

**Affiliations:** 1 College of Animal Science and Technology, Hainan University, Haikou 570228, China; simiao__zhao@163.com (S.Z.); cuihuiqi@webmail.hzau.edu.cn (H.C.); 20095200210007@hainanu.edu.cn (Z.H.); kych2008dl@163.com (L.D.); 2 College of Veterinary Medicine, Huazhong Agricultural University, Wuhan 430070, China

**Keywords:** Senecavirus A, GC content, adaptive evolution, evolutionary strategy, synonymous codon bias

## Abstract

Synonymous codon bias in the viral genome affects protein translation and gene expression, suggesting that the synonymous codon mutant plays an essential role in influencing virulence and evolution. However, how the recessive mutant form contributes to virus evolvability remains elusive. In this paper, we characterize how the Senecavirus A (SVA), a picornavirus, utilizes synonymous codon mutations to influence its evolution, resulting in the adaptive evolution of the virus to adverse environments. The phylogenetic tree and Median-joining (MJ)-Network of these SVA lineages worldwide were constructed to reveal SVA three-stage genetic development clusters. Furthermore, we analyzed the codon bias of the SVA genome of selected strains and found that SVA could increase the GC content of the third base of some amino acid synonymous codons to enhance the viral RNA adaptive evolution. Our results highlight the impact of recessive mutation of virus codon bias on the evolution of the SVA and uncover a previously underappreciated evolutionary strategy for SVA. They also underline the importance of understanding the genetic evolution of SVA and how SVA adapts to the adverse effects of external stress.

## 1. Introduction

Senecavirus A (SVA), originally known as Seneca Valley virus, is regarded as the only representative member of the genus Senecavirus in the family Picornaviridae [1]. SVA infection causes swine vesicular disease (SVD) and even death in newborn piglets, and has been prevalent worldwide since 2015 [2]. Before 2015, the severe, self-limited disease associated with SVA infection had been underestimated, partially because of the low incidence of infection, mild clinical symptoms, and local epidemics in Canada and the United States [3,4]. However, the incidence of diseases associated with various SVA mutants has increased in North America, South America, and Asia, including China, since 2015 [5,6,7,8,9]. Furthermore, swine infected by various mutants have shown severe clinical symptoms, and massive death has even affected newborn piglets, resulting in enormous economic losses in the trade of animals and animal products [10]. In recent years, the negative impact of SVA has been reevaluated owing to the high incidence and high variability rate of the SVA.

Scientists can reconstruct the viral genome with genetic engineering technology and then obtain the various attenuated strain vaccines to prevent numerous diseases associated with a given virus infection [11]. Unfortunately, few infectious diseases stemming from RNA viruses can be effectively controlled based on vaccination and antiviral treatment. For instance, during the influenza epidemic season, mutations, including antigenic drift and antigenic shift, should be monitored to study how viral genome changes lead to immune evasion elicited by vaccine immunization [12]. In general, the genome sequence of RNA viruses is of high genetic diversity, which is attributed to the deficiency of proofreading by RNA-dependent RNA polymerase during the virus replication, resulting in frequent virus RNA genome mutations [13,14]. Concurrently, frequent mutations are conducive to virus adaptability to changing environments [15], and genetic pressures by natural selection screen out the most adaptable genotype with phenotypic characteristics [16,17]. For example, McBride et al. demonstrated that the RNA virus φ6 can screen out highly adaptable genotypes for adaptation to the environment owing to its high mutation rate when it faces constant changes in the ambient temperature [18]. Such a mechanism allows the RNA virus to adapt to adverse environmental conditions, further maintaining the virulence of the virus. In addition to amino acid variation driven by self-beneficial mutations, synonymous codon mutations also contribute to genome evolution, although they have been considered silent, redundant, and meaningless mutations in the past [19]. Furthermore, several studies have discovered that codon bias affects protein translation efficiency, protein folding, transcription stability, fidelity, and gene expression [20,21,22,23,24]. The effects mentioned above may facilitate viruses to evade therapeutic drug interventions and resist the neutralizing antibodies induced by a vaccine. However, the current research on viral genome codons has primarily focused on how optimal codons affect protein expression and viral genome evolution [15,25].

This study analyzed 220 SVA sequences of viral samples during 1988–2018 from the GenBank. The phylogenetic tree of these SVA lineages across America and Asia was constructed to determine the phylogenetic grouping of SVA in genetic evolution. We also investigated Median-joining (MJ)–Network linear genetic evolution of the evolutionary model of SVA transmission. Based on fundamental data analysis of SVA codon bias, the primary genetic evolutionary pressure factors that may affect the codon bias of SVA were further determined. We observed an underestimated evolutionary strategy for promoting SVA adaptive evolution. Meanwhile, we hypothesized that this finding explains how evolutionary pressures by natural selection shape the adaptive trajectory of a virus species. To the best of our knowledge, this study used the most SVA gene sequences for the same type of study. We explored the evolutionary strategy of SVA through the variation rule of synonymous codon bias, revealing novel insights into the evolutionary dynamics of SVA, which can help tailor prevention and intervention strategies to facilitate the control of disease due to SVA infection.

## 2. Materials and Methods

### 2.1. Genome Sequence Acquisition of SVA

We downloaded a total of 220 complete genomic sequences of SVA from GenBank, which circulated in various countries between 1998–2018. Among them, 106 strains were isolated in the United States, 81 in China, 13 in Canada, 12 in Brazil, 6 in Thailand, 1 in Colombia, and 1 in Vietnam. The detailed sequence information is listed in Table S1.

### 2.2. Experimental Tools and Parameters

#### 2.2.1. Sequence Alignment and Phylogenetic Tree Construction

A total of 220 SVA complete genomic sequences were aligned using the Clustal W module in MEGA-X software. Given the influence of gene recombination events on the construction of phylogenetic trees and subsequent codon bias analysis [26,27], the recombination events of the selected sequences were evaluated and then implemented by RDP 5.0 software. Seven methods (RDP\Bootscan\Maxchi\GeneConv\Chimera\Siscan\3 Seq) were used to analyze recombination events during viral evolution. The SVA sequence was judged to be subject to a recombination event only when more than four of the seven methods represented simultaneous indicative markers for recombination. The strains with estimated recombination were excluded during the construction of the phylogenetic tree. The phylogenetic tree was constructed based on the maximum likelihood (ML) method using MEGA-X software, with a bootstrap value of 1000 replicates, and was visualized on the iTOL website. Then, MrBayes software was used to construct the Bayesian phylogenetic tree [28], which was visualized by Figtree v1.4.4 (http://tree.bio.ed.ac.uk/software/figtree (accessed on 25 March 2022)) software (Figure S1). Two algorithms were used for the phylogenetic analysis of virus strains to ensure its accuracy. 

#### 2.2.2. Construction of Median Joining Genetic Evolution Network Diagram

Phylogenetic network methods enable the visualization of many optimal trees, which can contribute to an understanding of virus evolution. Thus, the MJ-Network was constructed to discover the evolutionary relationships among SVA haplotypes using NETWORK 10.2.0.0 software [29]. The redundant strains were excluded, network analysis was performed based on the sequences of representative strains, and the MJ genetic evolution network diagram was constructed (Table S2).

### 2.3. Codon Bias Index and Analysis Method

DNASTAR software was used to analyze the entire gene sequence of representative strains to obtain the coding region sequence of the included strains. We converted the U base in the RNA sequence to the T base for software calculations. Next, the synonymous codon essential data were analyzed using CodonW 1.4 software and the Emboss online website CUSP module (Table S2). The essential data included the adequate number of codons (ENC), GC content in the coding region, GC content at the third codon positions of genes (GC3), and GC content at the first and second codon positions (GC12). Finally, statistical analysis and mapping were conducted using GraphPad Prism 8 software. The specific analysis using codon bias data was as follows.

#### 2.3.1. ENC and ENC Plot Analysis

ENC was used to determine codon bias indexes, which have widely been utilized to measure the strength of codon preference. The ENC value of each isolated strain was obtained by referring to and using the calculation method of the ENC value by Wright et al. [30]. An ENC value of 61 was obtained when all codons were used equally for each amino acid. A value of 20 was obtained for the ENC value when only one codon was used for each amino acid. A higher ENC value indicates low codon bias. Conversely, a lower ENC value indicates a more substantial codon usage bias while encoding the same amino acid.

ENC-Plot was used to analyze the codon usage bias factors [30]. In the ENC-Plot analysis, the GC3 content was used for the abscissa axis, and the ordinate was the value of ENC. In addition, when the mutation pressure determined the codon usage bias, the ENC value would be above the expected curve. Conversely, when the codon usage bias was mainly affected by natural selection and other factors rather than mutation pressure caused by codon bias, the ENC value would be below the expected standard curve. The calculation formula of the ENC expected standard curve was as follows [30] (where S represents the content of GC3):

$${\mathrm{ENC}}_{\mathrm{expected}}=2+S+(\frac{29}{s+{\left(1-s\right)}^{2}}).$$

#### 2.3.2. Neutrality Plot Analysis

Neutrality plot analysis is widely used to compare the influences of codon bias of mutation pressure and natural selection, and involves plotting the GC12 values of the synonymous codons against the GC3 values. Assuming that GC12 and GC3 have a significant correlation, and the linear regression curve slope is 1, this result would prove that the mutation pressure is the main influencing factor leading to the bias of synonymous codons. Conversely, there was no correlation between GC12 and GC3. The slope of the linear regression curve was close to 0, which indicates that natural selection played a dominant role in influencing codon bias, and the genes in the genome were highly conserved [31].

#### 2.3.3. Parity Rule 2 (PR2) Analysis

We performed PR2 analysis to assess whether mutation pressure or natural selection is the main influencing factor leading to changes in the codon usage pattern of the SVA genome. Using the abscissa A3/(A3 + T3) and the ordinate G3/(G3 + C3) to draw a scatter plot and a dividing line X = 0.5, Y = 0.5 in the figure, we calculated A3/(A3 + T3), G3/(G3 + C3) through A3, T3, G3, and C3. If mutation pressure and natural selection affected codon usage, these points would be at the dividing line, where A = U and G = C [32].

#### 2.3.4. SVA Encodes the Proportion of Synonymous Codons of the Same Protein

The coding region sequence of each SVA strain in the same cluster was integrated based on the cluster analysis result of the phylogenetic tree. Then, the integrated coding region sequence of different groups was obtained. The CUSP module in the EMBOSS website was used to calculate the proportion of synonymous codons when encoding the same amino acid for the integrated sequences under different clusters. Then, the ratio of synonymous codons was obtained using GraphPad Prism 8.0 software to draw a histogram, which encoded the same amino acid for the integrated sequences under different clusters. Subsequently, the scatter plot of the average proportion of these SVA synonymous codons encoding the same amino acid was drawn using GraphPad Prism 8.0 software.

### 2.4. Statistical Analysis

In this study, the sequence information of SVA strains was statistically sorted out by Microsoft Excel, and DNAstar software was used to sort out the coding region sequences of SVA strains. The basic data of SVA synonymous codons were analyzed by CodonW 1.4 software. In addition, the ENC, relative codon usage frequency and GC content in the analysis did not comply with normal distribution, and data were summarized by descriptive statistics. Univariate linear regression and correlation *p*-values were used in the neutrality plot analysis. A *p*-value of less than 0.05 was considered significant. Statistical analysis was performed using GraphPad Prism 8.0 software.

## 3. Results

### 3.1. Determination of Interspecific Recombination Events

Because recombination events interfere with the construction of phylogenetic trees and codon usage pattern analysis [33], we tested 220 SVA complete genomes from Gene Banks to analyze recombination events. A total of 33 SVA sequences were suspected as recombination events after three isolated strains were excluded, with significant sequence differences after alignment (Tables S1 and S3).

### 3.2. Phylogeny and MJ-Network Linear Genetic Evolution of SVA

**Figure 1:**
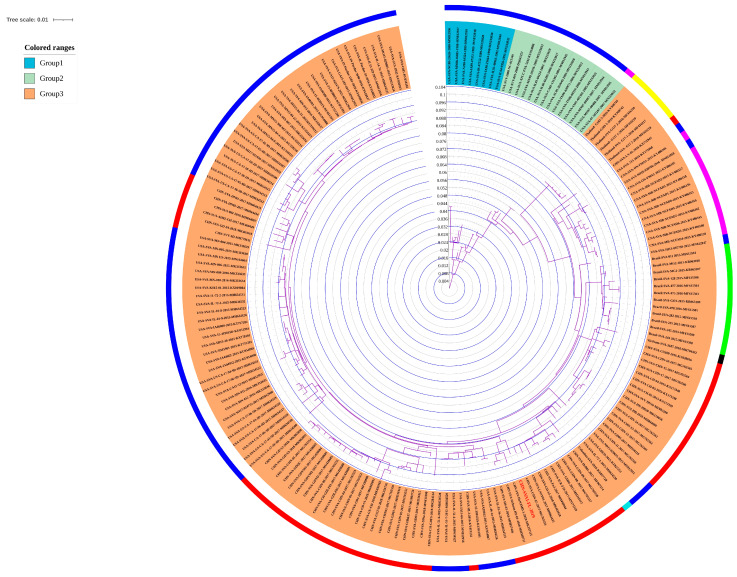
Phylogenetic tree analysis of SVA. The phylogenetic tree of SVA was constructed by the maximum likelihood calculation method. The inner-circle color fan represents the three genetic phylogenetic clusters among each strain of SVA. The phylogenetic groups 1, 2, and 3 are shown in blue, green, and orange. Concurrently, the solid round lines behind the gene’s name represent different countries: The United States is dark blue, China is red, Canada is purple, Thailand is yellow, Brazil is green, Colombia is light blue, and Vietnam is black.

The pathogenicity and infection rate of the virus showed an increasing trend, which indicates that SVA underwent a dramatic genetic variation compared with the strains isolated earlier. We constructed a phylogenetic tree of SVA without the suspected recombination event to evaluate the genetic evolution of SVA. The phylogenetic topology of the Bayesian tree (Figure S1) and ML tree were almost identical, and thus we chose the ML tree to be shown here (Figure 1). The results show that the clusters of the phylogenetic tree constructed had three major clusters, as shown in Figure 1. Furthermore, we found that with the isolation by the sequence of time as the primary basis, the viruses isolated between 1988–1993 gathered in the first group, the viruses isolated between 1995–2008 clustered in the second group, and the viruses isolated since 2008 were the third group (the currently circulating cluster). Moreover, the time limit of virus isolation and the evolutionary branches of the cluster also indicate the importance of geographical constraints on isolated strains for evolutionary clustering. For example, the strains isolated in Brazil were clustered in the same clade, and the time of virus isolation was the same period. Similarly, Thailand’s SVA, isolated in 2016, was also clustered in a smaller genetic branch.

**Figure 2:**
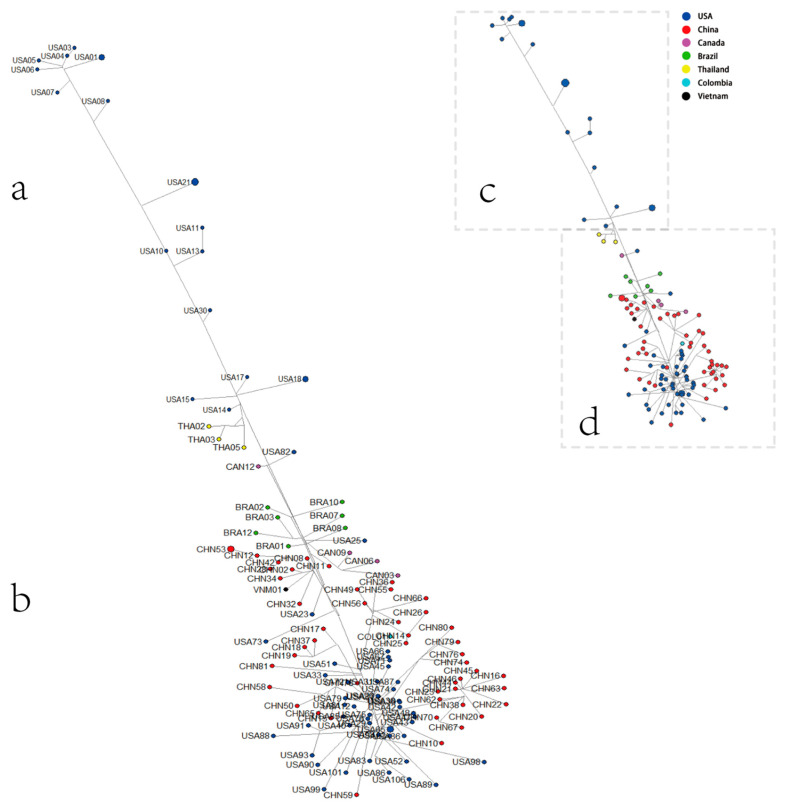
Median-joining (MJ)–Network diagram of SVA. The network diagram was constructed using MJ for the representative strains of SVA. Each scattered point represents an isolated SVA strain. (**a**) The top part is the distribution region of group I strains. (**b**) The upper part of the middle part is the distribution region of group II strains. The central and tail parts are the distribution region of group III strains. (**c**) A miniature diagram of (**a**). (**d**) A miniature diagram of (**b**).

The phylogenetic tree results indicate that SVA was divided into three clusters of genetic development stages based on the time and region during viral genetic evolution development. We performed an MJ-Network analysis to analyze and visualize the evolution of SVA and the route of the infection network and integrate our SVA isolates within this network. The results of the MJ-Network analysis show that the overall linear relationship was rough, of type “1,” as shown in Figure 2. We observed that the strains isolated in the United States in the late 1980s were in the upper part of the MJ-Network. The result suggests that the virus first originated in the United States, and since 2007, SVA has gradually spread in China, Thailand, Brazil, Vietnam, and other places. There is a trend of cross-spreading between countries with or without geographical restrictions, especially in the cross-spread between the provinces of China and the United States (Figure 2b).

### 3.3. The Codon Usage Pattern of the SVA

**Figure 3:**
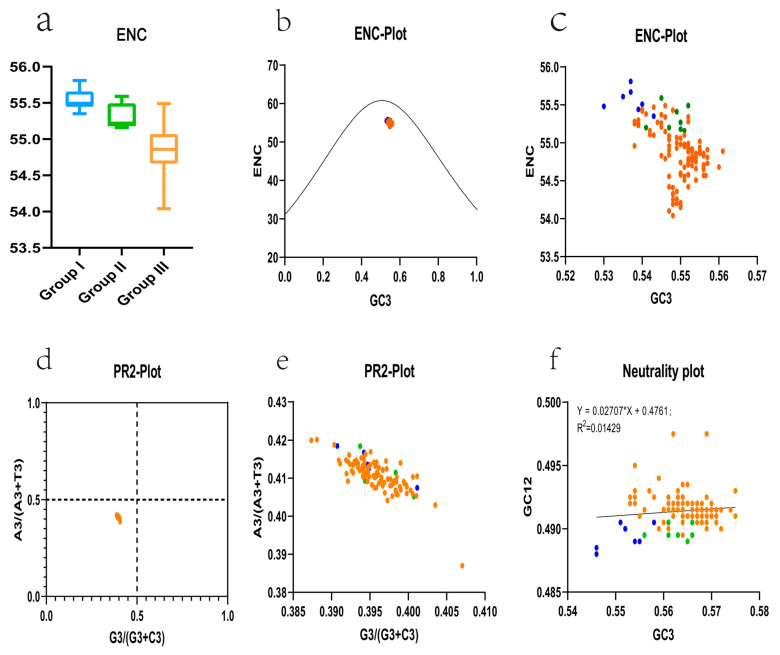
Codon bias analysis of the SVA. (**a**) The boxplot of ENC values of each strain under three groups. Group I, group II, and group III are represented by blue, green, and orange, respectively. (**b**) The ENC-plot of the SVA represents the relationship between the ENC value and GC3 of each strain. The scattered points of ENC and GC3 of all strains were distributed uniformly below the standard curve and were relatively concentrated. (**c**) A locally enlarged view of (**b**). Each scatter strain represents the result of an ENC-Plot analysis of an SVA strain. The blue scatter represents strains from group I, the green scatter represents strains from group II, and the orange scatter represents strains from group III. (**d**) PR2-plot analysis of SVA coding region sequences distributed under different genetic clusters. All SVA strain PR2-plot scatters were distributed in the lower-left region and were relatively concentrated. (**e**) A locally enlarged view of (**d**). (**f**) Neutrality plot analysis (GC12 vs. GC3) of SVA. GC12 stands for the average value of GC contents at the first and second positions of the codons (GC1 and GC2), while GC3 refers to the GC contents at the third codon position. The black straight line represents the regression line. The regression equation is also shown.

We analyzed the usage patterns of synonymous codons in the coding region of the SVA genome to elucidate the rule of selection pressures on the evolution of SVA. The adequate codon number of each SVA strain, namely the ENC value, was measured, and we found that the ENC of each strain was relatively concentrated, ranging from 54.04 to 55.81 (Table S2). The ENC values of all SVA strains were higher than the 35 critical values, which indicates that there was no apparent bias in the overall codon usage of SVA, and the frequency of synonymous codon usage was relatively balanced. In addition, the distribution range of ENC values of each SVA strain showed a negative correlation with the time of strain isolation (Figure 3a). The above results indicate that the recent and modern pandemic strains showed lower ENC values, which reveals a trend toward a greater preference for using synonymous codons compared with strains isolated in early strains in the United States.

We used ENC-plot analysis to investigate what kind of selection pressure affected codon usage patterns. The results show that all the scattered points were evenly distributed below the standard curve, indicating that the natural selection pressure affects the SVA codon bias (Figure 3b). Furthermore, by enlarging Figure 3b, we observed that the first cluster of early United States strains was closer to the standard curve, while the currently circulating strains tended to move downward, away from the standard curve (Figure 3c). In addition, PR2 analysis and neutral mapping analysis were performed further to identify the influencing factors of codon usage bias. The PR2 analysis results show that all scattered points of SVA PR2 were located in the lower-left region of the figure (Figure 3d,e), which suggests that the composition of the third codon base in the triad codon of the SVA virus coding region is biased. The number of pyrimidines is higher than the number of purines. Furthermore, the neutrality plot analysis results show no significant correlation between the GC content of the third position in the triplet codon of the coding region of the viral genome. The GC content of the first and second positions (*p* = 0.205) and the slope of the linear regression curve was less than 0.5, which is closer to 0 (Figure 3f). These data indicate that natural selection pressures play a dominant role in codon bias. At the same time, genetic drift has a negligible effect on the codon bias of the SVA typical strains selected in our analysis.

### 3.4. Trend Change of GC Content in SVA Coding Region

We analyzed the variation trend of GC content in the SVA coding region at various stages of genetic evolution. According to the previously established SVA phylogenetic tree, the evolutionary history of SVA was divided into three clusters of genetic development stages (Figure 1). Then, we analyzed the average proportions of GC content in the coding regions of SVA during these three stages of genetic evolution. The GC content with coding regions showed that the average GC content of the original strain cluster was 51.03%, the moderate GC content of the intermediate transition cluster was 51.37%, and the average GC content of the current circulating strain cluster was 51.61% (Figure S2). These data suggest that with the evolution of SVA, GC content in its coding region has an increasing trend.

### 3.5. Content of GC in the Third Base of the Synonymous Codon

**Figure 4:**
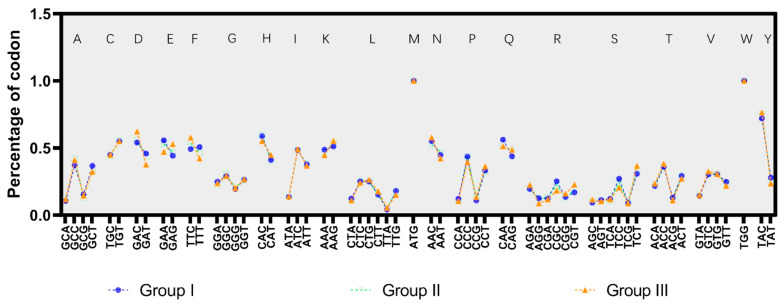
Quantitative indicators of changes in synonymous codon usage patterns of amino acids during the evolution of the SVA. Each scattered point represents the usage frequency of synonymous codon use, blue represents the usage frequency of group I strains, green represents the usage frequency of group II, and orange represents group III strains. The 20 amino acid names are denoted by abbreviated letters.

To explore the evolution of SVA synonymous codon bias, we analyzed the indices of the changes in the utilization frequency of the synonymous codons of each amino acid at different stages of SVA genetic evolution. The sequences of coding regions integrated all strains in each cluster to obtain three gene sets representing the three genetic evolution clusters of SVA. We detected differences in the relative frequency of synonymous codons when encoding the same amino acid in different clusters by probing the synonymous codon utilization frequency in three gene sets (as shown in Figure 4 and Table S4).

Figure 4 shows that the top three amino acids with the most significant changes were phenylalanine, glutamate, and aspartic acid. We compared the usage frequency of the phenylalanine, glutamate, and aspartic acid synonymous codons in the three genetic evolutionary clusters of SVA. The TTC of phenylalanine synonymous codon increased from 49.3% of the original strain to 57.8% of the current epidemic strain, the GAG of glutamate synonymous codon increased from 44.3% to 52.9%, and the GAC of aspartic acid synonymous codon rose from 54.1% to 62.3% (Table S4). The comparison results show that the third base of some amino acid synonymous codons follows the rule that the GC content of the third base of the amino acid synonymous codon gradually increases more in the currently circulating cluster compared to the origin cluster.

## 4. Discussion

One of the key advantages of virus survival is their adaptive evolution, which allows individuals in the population to better cope with the challenges posed by the current environment [34]. We reported on a synonymous codon evolution strategy for the Picornaviridae family member SVA for the first time. The analysis based on SVA synonymous codon correlation showed that the change trend of GC content of SVA synonymous codons. Our results highlight the impact of recessive mutations of virus codon bias on the evolution of the virus and point to a previously underappreciated evolutionary strategy for viruses to promote adaptive evolution.

It has been a fundamental expectation of evolutionary biology, as well as for the evolution of viruses, that the dynamics of molecular adaptation can be understood [35]. RNA viruses accumulate genetic variation rapidly, owing to their replication via an RNA-dependent RNA polymerase (RdRp) and short generation time. As a result of the specificity of replication, they have a large population size and genetic diversity, which enables them to adapt quickly to environmental changes [36]. For a species, a beneficial mutation is the basis of adaptive evolution driven by natural selection, whereas for a beneficial mutation to be fixed in a population, it must be survived by many mutations and replaced by a selection in the rest of the genome [37]. 

The development of high-throughput sequencing technology enabled us to perform phylogenetic analysis on complete genomes obtained at various stages of the evolutionary series. This allowed us to group them into clusters that represented genetic stages in their genetic evolution [38]. However, there are many limitations on obtaining an accurate phylogenetic tree that can reflect the most realistic evolutionary history of species. Analysis of the sample number, nucleotide length, and selection of calculation method for phylogenetic tree construction are the key factors to ensure the accuracy of the phylogenetic tree [39,40]. In this study, we avoided the influence of the length of nucleotides examined and the number of samples on the accuracy of constructing the phylogenetic tree. Note that the results of the MJ-Network analysis (Figure 2b) indicate that strains in the current epidemic phase are being explored through different adaptive evolution, ultimately evolving in a favorable direction to adapt to the environment and host so as to increase selective evolutionary pressure.

Note that although RNA viruses have extremely high mutation rates, they do not mutate unrestrained. It has been recognized that the strong evolutionary constraints on RNA viral genomes are reflected in the distribution of mutational adaptation effects of RNA viruses [41]. Moreover, natural selection acts on the phenotypic diversity of mutant genomes in a population to push the population toward increased fitness [25]. What is interesting is that evolution at this point has a dynamic balance that is both contradictory and harmonious, and this balance is guaranteed by genetic robustness [42]. Then, genetic robustness comes into play when a species reaches a dominant genome, ensuring the sustainable development of adaptive evolution [43]. Moreover, the role of codons in promoting viruses has been widely studied by reviewing genetic robustness [44]. Codon bias is critical for maximizing viral robustness by limiting the possibility of harmful mutations [25]. The ENC value of SVA in this study is in agreement with the report by Chen Y et al. [45]. Additionally, the ENC value of SVA also conforms to the range of 38.9–58.3 average ENC value of RNA virus, as previously reported. [46]. Of note, the ENC value of the current SVA circulating strains decreased compared to the strains isolated earlier. It also suggests that the use of SVA codons shows a more adaptive evolutionary bias toward codon usage. Furthermore, the evolutionary pressure of natural selection is the main factor that causes codon bias in various SVA strains. Similarly, natural selection pressure also affects codon bias of the genome in Enterovirus 71, Cardiovirus, and Foot-and-mouth disease virus in the family Picornaviridae [47,48], indicating that the fluctuation of the replication environment of an RNA virus is the most important factor to drive virus mutation.

As the GC content of the genome has been demonstrated to be the main factor of codon usage bias, the codon bias of RNA viruses is mainly driven by GC content [49]. Fortunately, the genomic differences of SVA under different genetic clusters were revealed by our analysis. Notably, high GC content has many evolutionary benefits for the virus. One of the benefits is that high GC content can produce stable RNA secondary structures. In the long neck-chain formed by RNA secondary structures, the GC base pair acquires three hydrogen bonds, which contribute to the structural stability of RNA secondary structures [50,51]. Studies have shown that the genome temperature optima and radiation resistance positively correlate with GC content owing to improved RNA structural stability [52,53]. Another benefit of increased GC content is that it promotes mRNA stability. As a parasitic microorganism in the host cell, RNA viruses compete with host cells for translation and expression at the mRNA level [54]. Improving viral mRNA stability helps the virus resist the effects of host cell RNA decay mechanisms on viral mRNA [55]. Hia et al. confirmed that codon bias is a key mechanism regulating RNA stability and demonstrated that the contents of GC and GC3 are positively correlated with mRNA stability [56]. In addition, Gelfman et al. found that one benefit of high GC content is that it can enhance the flexible sexual area of the genome, which may promote the nucleosome combination [57]. Nucleosome localization plays an important role in gene expression regulation [58]. Currently, the COVID-19 virus poses a serious threat to human health. Li et al. studied the GC content of COVID-19 and the adaptability of the human lung environment, and the results also confirm that the benefits provided by high GC content include the enhanced adaptability of the virus [59].

Additionally, small interfering RNAs (siRNAs) are crucial for host defense against viruses, which can inhibit or silence the expression of abnormal and foreign genes [60,61]. Studies have shown that high GC content can inhibit siRNAs’ interference effect, and transgenic high GC content also has shown higher transcription and protein accumulation [62]. Overall, the increased GC content in the genome benefits the replication of the virus, thus improving the resistance of SVA to the challenge of adverse external conditions. The role of this recessive mutation form of synonymous codon bias was again demonstrated by our data analysis. Our findings suggest that the Picornaviridae family member SVA codon bias evolution strategy plays another role in the evolution of the virus.

Moreover, we speculate that the evolutionary trend of codon bias we found is one of the reasons for the increased harmfulness of SVA. Our study revealed the evolutionary strategy of the SVA genome from the perspective of recessive mutation, which is easily ignored. Our findings are expected to provide a scientific theoretical basis for optimizing SVA synonymous codons and exploring SVA dominant evolutionary strategies.

Notably, this study had several limitations. First, in analyzing the increase in GC content in the third base of the SVA synonymous codon and its adaptive evolution, we found that not all the third bases of the amino acid synonymous codon followed the trend of GC content increase. As shown in Figure 4, the evolution trend of the synonymous codon of histidine and arginine showed a decreasing trend of GC content at the third base. We suspect that the codon has a strong bias when the host encodes the above amino acids. The virus makes a clever concession to avoid the head-on and vicious competition with the host regarding tRNA abundance for the viral replication efficiency [63]. Therefore, our data only demonstrate that during the genetic evolution of SVA, in the SVA coding part but not all amino acids, its synonymous codon follows the law of increasing the proportion of GC content in the third base. Second, we did not demonstrate that the current population of SVA-prevalent strains was a cluster of the most adaptive dominant species during its genetic evolution. There is no substantial evidence that the currently circulating cluster is more robust in adaptability, virulence, and transmission ability than the earlier isolated strain. It cannot be ruled out that people did not pay enough attention to SVA in the early stage or mistook the clinical symptoms caused by SVA for other pathogens, which may have led to incomplete or under-reported cases of SVA. Therefore, it is not rigorous or scientific to infer from existing epidemiological reports of SVA that current SVA strains have increased resistance to external adverse factors compared to earlier isolated SVA strains. At present, reverse genetic technology is widely used to construct full-length infectious clones of virus strains, so it can be used to construct full-length infectious clones of early isolates strains. We also analyzed differences in virulence and temperature sensitivity between the early isolates and the prevalent cluster strains. We believe that the analysis results can help us better understand the evolutionary strategy of SVA viruses.

## Data Availability

Not applicable.

## Supplementary Materials

Supporting information can be downloaded at: https://www.mdpi.com/article/10.3390/v14051055/s1, Figure S1: The phylogenetic tree of SVA constructed by the Bayesian algorithm; Figure S2: Histogram of the GC content of each strain in the SVA coding region; Table S1: Sequence information of SVA strains; Table S2: Codon bias calculation data of SVA strains; Table S3: Information on recombination events of the isolated SVA; Table S4: Quantitative index of changes in synonymous codon usage patterns of amino acids in three groups of SVA.
