# Supplementary material for: Senecavirus A Enhances Its Adaptive Evolution via Synonymous Codon Bias Evolution
Source: Viruses. 2022 May 16;14(5):1055. doi: 10.3390/v14051055 (PMC9146685; doi:10.3390/v14051055)
Supplement: Supplementary file 1 [file viruses-14-01055-s001.zip › Figure S1- The phylogenetic tree of Senecavirus A is constructed by Bayesian algorithm..pdf]

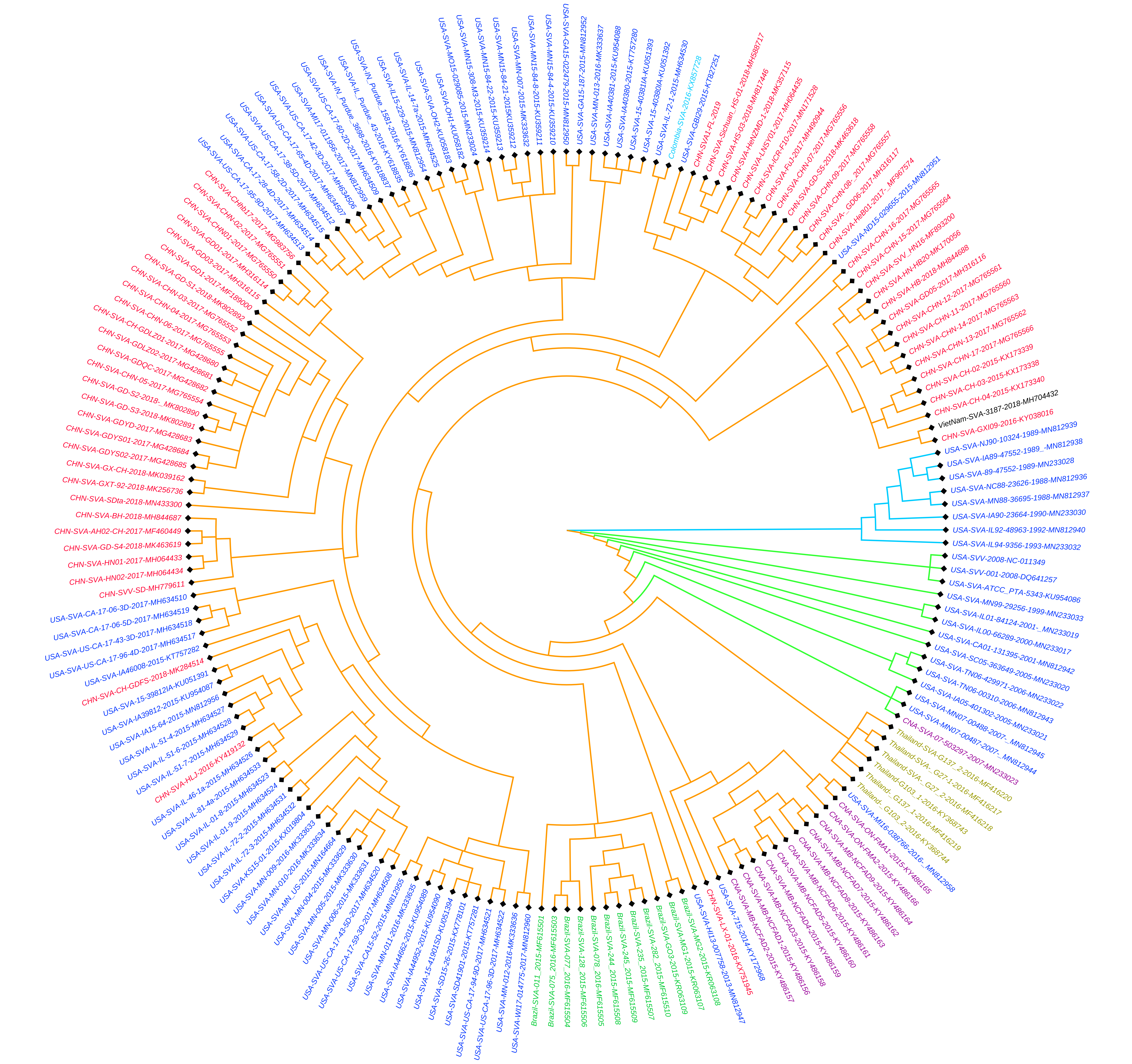
USA-SVA-OH1-KU058182
USA-SVA-OH2-KU058183
USA-SVA-IL-14-7a-2015-MH634525
USA-SVA-IL15-229-2015-MN812954
USA-SVA-IN_Purdue_1581-2016-KY618836
USA-SVA-IL_Purdue_43-2016-KY618835
USA-SVA-IN_Purdue_3698-2016-KY618837
USA-SVA-US-CA-17-60-2D-2017-MH634509
USA-SVA-MI17-011956-2017-MN812959
USA-SVA-US-CA-17-42-3D-2017-MH634506
USA-SVA-US-CA-17-65-4D-2017-MH634507
USA-SVA-US-CA-17-38-5D-2017-MH634512
USA-SVA-US-CA-17-58-2D-2017-MH634515
USA-SVA-CA-17-28-4D-2017-MH634514
USA-SVA-US-CA-17-95-9D-2017-MH634513
CHN-SVA-CHhb17-2017-MG983756
CHN-SVA-CHN-02-2017-MG765551
CHN-SVA-CHN01-2017-MG765550
CHN-SVA-GD01-2017-MH316114
CHN-SVA-GD03-2017-MH316115
CHN-SVA-GD1-2017-MF189000
CHN-SVA-GD-S1-2018-MK802892
CHN-SVA-CHN-03-2017-MG765552
CHN-SVA-CHN-04-2017-MG765553
CHN-SVA-CHN-06-2017-MG765555
CHN-SVA-CH-GDLZ01-2017-MG428680
CHN-SVA-GDLZ02-2017-MG428681
CHN-SVA-GDQC-2017-MG428682
CHN-SVA-CHN-05-2017-MG765554
CHN-SVA-GD-S2-2018-_MK802890
CHN-SVA-GD-S3-2018-MK802891
CHN-SVA-GDYD-2017-MG428683
CHN-SVA-GDYS01-2017-MG428684
CHN-SVA-GDYS02-2017-MG428685
CHN-SVA-GX-CH-2018-MK039162
CHN-SVA-GXT-92-2018-MK256736
CHN-SVA-SDta-2018-MN433300
CHN-SVA-BH-2018-MH844687
CHN-SVA-AH02-CH-2017-MF460449
CHN-SVA-GD-S4-2018-MK463619
CHN-SVA-HN01-2017-MH064433
CHN-SVA-HN02-2017-MH064434
CHN-SVV-SD-MH779611
USA-SVA-CA-17-06-3D-2017-MH634510
USA-SVA-CA-17-06-5D-2017-MH634519
USA-SVA-US-CA-17-43-3D-2017-MH634518
USA-SVA-US-CA-17-96-4D-2017-MH634517
USA-SVA-IA46008-2015-KT757282
CHN-SVA-CH-GDFS-2018-MK284514
USA-SVA-15-39812IA-KU051391
USA-SVA-IA39812-2015-KU954087
USA-SVA-IA15-64-2015-MN812956
USA-SVA-IL-51-4-2015-MH634527
USA-SVA-IL-51-6-2015-MH634528
USA-SVA-IL-51-7-2015-MH634529
CHN-SVA-HLJ-2016-KY419132
USA-SVA-IL-46-1a-2015-MH634526
USA-SVA-IL-81-4a-2015-MH634533
USA-SVA-IL-01-8-2015-MH634523
USA-SVA-IL-01-9-2015-MH634524
USA-SVA-IL-72-2-2015-MH634531
USA-SVA-IL-72-3-2015-MH634532
USA-SVA-KS15-01-2015-KX019804
USA-SVA-MN-009-2016-MK333633
USA-SVA-MN-010-2016-MK333634
USA-SVA-MN_US-2015-MN164664
USA-SVA-MN-004-2015-MK333629
USA-SVA-MN-005-2015-MK333630
USA-SVA-MN-006-2015-MK333631
USA-SVA-US-CA-17-43-5D-2017-MH634520
USA-SVA-US-CA-17-59-3D-2017-MH634508
USA-SVA-CA15-52-2015-MN812955
USA-SVA-MN-011-2016-MK333635
USA-SVA-IA44662-2015-KU954089
USA-SVA-IA44952-2015-KU954090
USA-SVA-15-41901SD-KU051394
USA-SVA-SD15-26-2015-KX778101
USA-SVA-SD41901-2015-KT757281
USA-SVA-US-CA-17-94-9D-2017-MH634521
USA-SVA-US-CA-17-96-3D-2017-MH634522
USA-SVA-MN-012-2016-MK333636
USA-SVA-WI17-014775-2017-MN812960
Brazil-SVA-011_2015-MF615501
Brazil-SVA-075_2016-MF615503
Brazil-SVA-077_2016-MF615504
Brazil-SVA-128_2015-MF615506
Brazil-SVA-078_2016-MF615505
Brazil-SVA-244_2015-MF615508
Brazil-SVA-245_2015-MF615509
Brazil-SVA-235_2015-MF615507
Brazil-SVA-282_2015-MF615510
Brazil-SVA-GO3-2015-KR063109
Brazil-SVA-MG1-2015-KR063107
Brazil-SVA-MG2-2015-KR063108
USA-SVA-HI13-007758-2013-MN812947
CHN-SVA-LX-01-2016-KX751945
USA-SVA-715-2014-KY172968
CNA-SVA-MB-NCFAD2-2015-KY486157
CNA-SVA-MB-NCFAD1-2015-KY486156
CNA-SVA-MB-NCFAD3-2015-KY486158
CNA-SVA-MB-NCFAD4-2015-KY486159
CNA-SVA-MB-NCFAD5-2015-KY486160
CNA-SVA-MB-NCFAD6-2015-KY486161
CNA-SVA-MB-NCFAD8-2015-KY486162
CNA-SVA-MB-NCFAD7-2015-KY486163
CNA-SVA-MB-NCFAD9-2015-KY486164
CNA-SVA-ON-FMA2-2015-KY486166
CNA-SVA-ON-FMA1-2015-KY486165
USA-SVA-MI16-038766-2016_-MN812958
Thailand-_G103_2-2016-KY368744
Thailand-_G137_1-2016-MF416219
Thailand-G103_1-2016-KY368743
Thailand-SVA-_G27_2-2016-MF416218
Thailand-SVA-_G27_1-2016-MF416217
Thailand-SVA-G137_2-2016-MF416220
CNA-SVA-07-503297-2007-MN233023
USA-SVA-MN07-00487-2007-_MN812944
USA-SVA-MN07-00488-2007-_MN812945
USA-SVA-IA05-401302-2005-MN233021
USA-SVA-TN06-00310-2006-MN812943
USA-SVA-TN06-429971-2006-MN233022
USA-SVA-SC05-363649-2005-MN233020
USA-SVA-CA01-131395-2001-MN812942
USA-SVA-IL00-66289-2000-MN233017
USA-SVA-IL01-84124-2001-_MN233019
USA-SVA-MN99-29256-1999-MN233033
USA-SVA-ATCC_PTA-5343-KU954086
USA-SVV-001-2008-DQ641257
USA-SVV-2008-NC-011349
USA-SVA-IL94-9356-1993-MN233032
USA-SVA-IL92-48963-1992-MN812940
USA-SVA-IA90-23664-1990-MN233030
USA-SVA-MN88-36695-1988-MN812937
USA-SVA-NC88-23626-1988-MN812936
USA-SVA-89-47552-1989-MN233028
USA-SVA-IA89-47552-1989_-MN812938
USA-SVA-NJ90-10324-1989-MN812939
CHN-SVA-GXI09-2016-KY038016
VietNam-SVA-3187-2018-MH704432
CHN-SVA-CH-04-2015-KX173340
CHN-SVA-CH-03-2015-KX173338
CHN-SVA-CH-02-2015-KX173339
CHN-SVA-CHN-17-2017-MG765566
CHN-SVA-CHN-13-2017-MG765562
CHN-SVA-CHN-14-2017-MG765563
CHN-SVA-CHN-11-2017-MG765560
CHN-SVA-CHN-12-2017-MG765561
CHN-SVA-GD05-2017-MH316116
CHN-SVA-HB-2018-MH844688
CHN-SVA-HN-HB20-MK170056
CHN-SVA-SVV_HN16-MF893200
CHN-SVA-CHN-15-2017-MG765564
CHN-SVA-CHN-16-2017-MG765565
USA-SVA-ND15-029655-2015-MN812951
CHN-SVA-HeB01-2017-_MF967574
CHN-SVA-_GD06-2017-MH316117
CHN-SVA-CHN-08_2017-MG765557
CHN-SVA-CHN-09-2017-MG765558
CHN-SVA-GD-S5-2018-MK463618
CHN-SVA-CHN-07-2017-MG765556
CHN-SVA-FuJ-2017-MH490944
CHN-SVA-ICR-F10-2017-MN171528
CHN-SVA-LNSY01-2017-MH064435
CHN-SVA-HeNZMD-1-2018-MK357115
CHN-SVA-HS-03-2018-MH817446
CHN-SVA-Sichuan_HS-01-2018-MH588717
CHN-SVA1-FJ-2019
USA-SVA-GBI29-2015-KT827251
Colombia-SVA-2016-KX857728
USA-SVA-IL-72-1-2015-MH634530
USA-SVA-15-40380IA-KU051392
USA-SVA-15-40381IA-KU051393
USA-SVA-IA40380-2015-KT757280
USA-SVA-IA40381-2015-KU954088
USA-SVA-MN-013-2016-MK333637
USA-SVA-GA15-187-2015-MN812952
USA-SVA-GA15-022479-2015-MN812950
USA-SVA-MN15-84-4-2015-KU359210
USA-SVA-MN15-84-8-2015-KU359211
USA-SVA-MN-007-2015-MK333632
USA-SVA-MN15-84-21-2015KU359212
USA-SVA-MN15-84-22-2015-KU359213
USA-SVA-MN15-308-M3-2015-KU359214
USA-SVA-MO15-029085-2015-MN233024

Supplementary Figure Legends

Figure S1. The phylogenetic tree of SVA is constructed by a Bayesian algorithm

The phylogenetic tree of SVA was constructed by the Bayesian calculation method. The color of the branching lines in the inner circle represents the phylogenetic clusters of the SVA, and here we use Group I, Group II, and Group III in blue, green, and saffron yellow. The color of the name of the round outer strain indicates the country from which it was isolated. Here, we use dark blue for the United States, red for China, purple for Canada, yellow for Thailand, green for Brazil, light blue for Colombia, and black for Vietnam.
