# Supplementary material for: Senecavirus A Enhances Its Adaptive Evolution via Synonymous Codon Bias Evolution
Source: Viruses. 2022 May 16;14(5):1055. doi: 10.3390/v14051055 (PMC9146685; doi:10.3390/v14051055)
Supplement: Supplementary file 1 [file viruses-14-01055-s001.zip › Figure S2-Histogram of GC content of each strain in SVA coding region.pdf]

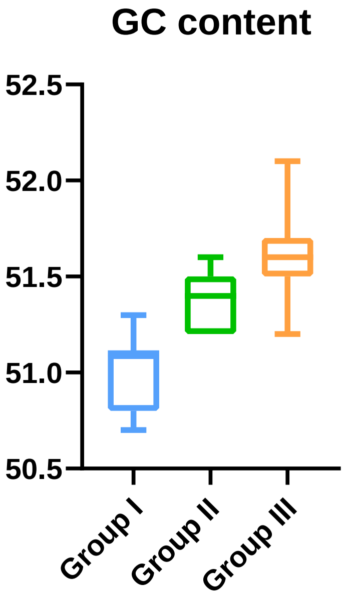
GC content
52.5
52.0
51.5
51.0
50.5
Group I
Group II
Group III

Figure S2. Histogram of the GC content of each strain in the SVA coding region

The histogram shows the percentage of the GC content of each SVA strain in the coding region under different clusters, where Group I represents the early isolated strains and is shown in blue, and Group II means the intermediate transitional strains. It is shown in green. Group III represents the current prevalent strains and is shown in orange. The content of GC in the SVA coding region showed an increasing trend.
