# Supplementary material for: Senecavirus A Enhances Its Adaptive Evolution via Synonymous Codon Bias Evolution
Source: Viruses. 2022 May 16;14(5):1055. doi: 10.3390/v14051055 (PMC9146685; doi:10.3390/v14051055)
Supplement: Supplementary file 1 [file viruses-14-01055-s001.zip › Table S3- Information of recombination events of the isolated SVA.pdf]

Supplementary Table S3. Information on recombination events of the SVA tested in the study

Event number	Abbreviate virus strain	Major parent	Minor parent	Breakpoints (In alignment)		Average p-value of the detection methods						
				Beginning	Ending	RDP	Bootscan	Maxchi	GeneConv	Chimera	Siscan	3Seq
1	USA-09	USA-24	USA-78	6987	2770	ND	ND	2.659×10^{-5}	ND	4.233×10^{-4}	8.763×10^{-3}	4.224×10^{-2}
2	USA-22	USA-24	Unknown	1590	3434	4.555×10^{-6}	6.578×10^{-3}	1.491×10^{-7}	ND	7.470×10^{-8}	7.262×10^{-6}	1.299×10^{-9}
3	CAN-02	CAN-05	Unknown	3060	7114	ND	ND	2.708×10^{-5}	ND	1.437×10^{-3}	1.425×10^{-6}	3.248×10^{-10}
4	USA-24	USA-51	CAN-04	2110	3580	NS	ND	2.027×10^{-3}	ND	1.342×10^{-3}	1.956×10^{-2}	1.686×10^{-3}
5	CHN-01	CHN-13	CHN-04	17	1440	5.295×10^{-11}	1.748×10^{-7}	2.160×10^{-3}	1.380×10^{-6}	5.131×10^{-3}	1.162×10^{-4}	5.251×10^{-9}
6	CHN-05	CHN-09	Unknown	17	1687	1.097×10^{-10}	1.487×10^{-6}	3.743×10^{-4}	3.741×10^{-7}	2.374×10^{-3}	1.027×10^{-2}	4.907×10^{-8}
7	USA-35	USA-42	CHN-16	892	1808	6.807×10^{-2}	ND	2.906×10^{-3}	ND	1.105×10^{-2}	ND	1.926×10^{-3}
8	USA-55	USA-58	CHN-56	1292	1828	9.707×10^{-5}	1.194×10^{-3}	ND	5.533×10^{-4}	ND	ND	1.896×10^{-2}
9	USA-56	USA-70	Unknown	1101	3048	4.753×10^{-2}	ND	9.922×10^{-4}	1.268×10^{-3}	ND	3.958×10^{-15}	1.216×10^{-3}
10	USA-57	USA-70	Unknown	866	3076	1.863×10^{-2}	ND	1.539×10^{-4}	ND	1.450×10^{-5}	ND	1.502×10^{-5}
11	USA-59	USA-87	Unknown	1239	3048	2.593×10^{-2}	ND	6.005×10^{-8}	1.096×10^{-3}	7.135×10^{-6}	1.984×10^{-12}	1.406×10^{-5}
12	USA-60	USA-58	CHN-56	1238	1968	7.264×10^{-5}	8.176×10^{-5}	ND	4.141×10^{-4}	ND	ND	2.406×10^{-2}
13	USA-61	CHN-68	Unknown	2312	5118	ND	ND	9.925×10^{-6}	ND	6.455×10^{-3}	7.672×10^{-3}	1.841×10^{-5}
14	CHN-06	CHN-19	CHN-56	7012	3570	2.513×10^{-3}	ND	4.564×10^{-6}	ND	1.098×10^{-4}	1.468×10^{-9}	6.077×10^{-10}
15	CHN-09	CHN-13	CHN-04	7070	1440	6.016×10^{-11}	1.986×10^{-7}	1.452×10^{-3}	1.569×10^{-6}	5.830×10^{-3}	1.320×10^{-4}	5.966×10^{-9}
16	CHN-13	CHN-09	Unknown	7070	1440	1.097×10^{-10}	1.487×10^{-6}	3.743×10^{-4}	3.741×10^{-7}	2.374×10^{-3}	1.027×10^{-2}	4.907×10^{-8}
17	CHN-27	CHN-28	CHN-19	4021	5992	1.285×10^{-8}	6.676×10^{-5}	1.344×10^{-7}	1.125×10^{-6}	2.821×10^{-7}	8.745×10^{-6}	9.446×10^{-12}
18	CHN-35	USA-41	CHN-25	1250	2048	5.700×10^{-3}	9.985×10^{-3}	1.855×10^{-2}	ND	1.109×10^{-2}	ND	1.666×10^{-3}
19	CHN-39	CHN-38	CHN-35	3319	4512	ND	ND	2.107×10^{-6}	2.558×10^{-11}	2.064×10^{-6}	1.818×10^{-7}	1.945×10^{-16}
20	CHN-48	USA-85	CHN-09	7070	816	8.887×10^{-6}	ND	ND	3.397×10^{-4}	ND	1.498×10^{-2}	1.609×10^{-2}
21	CHN-52	USA-41	CHN-25	1250	2048	2.577×10^{-3}	4.641×10^{-3}	2.744×10^{-2}	ND	1.072×10^{-2}	4.475×10^{-2}	1.400×10^{-3}
22	CHN-54	USA-41	CHN-25	1250	2070	2.313×10^{-3}	7.009×10^{-3}	2.333×10^{-2}	ND	9.120×10^{-3}	ND	1.190×10^{-3}
23	USA-96	USA-41	USA-60	842	2656	5.483×10^{-3}	ND	3.280×10^{-3}	ND	8.674×10^{-4}	1.717×10^{-2}	3.686×10^{-6}
24	USA-105	USA-41	CHN-16	855	2467	1.103×10^{-3}	8.139×10^{-3}	1.874×10^{-3}	ND	2.612×10^{-4}	ND	6.489×10^{-5}

25	CHN-57	CHN-40	Unknown	2132	3482	1.785×10^{-4}	ND	2.200×10^{-6}	ND	5.430×10^{-7}	3.771×10^{-8}	2.079×10^{-9}
26	CHN-60	CHN-24	CHN-19	4245	5844	7.241×10^{-8}	ND	1.959×10^{-3}	3.373×10^{-2}	3.673×10^{-4}	7.738×10^{-5}	1.247×10^{-8}
27	CHN-61	USA-41	CHN-56	852	2041	5.148×10^{-4}	1.165×10^{-2}	1.416×10^{-2}	ND	4.762×10^{-3}	ND	8.711×10^{-3}
28	CHN-68	USA-41	CHN-25	1250	2048	2.855×10^{-2}	ND	2.076×10^{-2}	ND	1.085×10^{-2}	ND	1.999×10^{-3}
29	CHN-69	USA-41	CHN-25	1250	2069	2.855×10^{-2}	ND	2.076×10^{-2}	ND	1.805×10^{-2}	ND	1.999×10^{-3}
30	CHN-72	CHN-58	CHN-74	4086	5702	1.676×10^{-11}	2.146×10^{-2}	2.447×10^{-7}	8.545×10^{-9}	6.475×10^{-6}	3.728×10^{-10}	3.251×10^{-5}
31	CHN-73	CHN-58	CHN-74	4077	5702	1.421×10^{-12}	4.470×10^{-7}	3.761×10^{-7}	3.988×10^{-10}	3.888×10^{-6}	4.384×10^{-10}	4.985×10^{-8}
32	CHN-75	USA-41	CHN-25	1250	1974	2.855×10^{-2}	ND	2.076×10^{-2}	ND	1.805×10^{-2}	ND	1.999×10^{-3}
33	CHN-77	USA-41	CHN-25	1250	2048	2.855×10^{-2}	ND	2.076×10^{-2}	ND	1.805×10^{-2}	ND	1.999×10^{-3}

ND: Recombination not detected with this algorithm.

In alignment: The genome sequence has been aligned by the alignment correction module of the Mega-X software.

Abbreviate: The full name of each virus strain has been abbreviated in this study. The exact full name is shown in Supplementary Table 1.
